# Nanobased Natural Polymers as a Carrier System for Glyphosate: An Interesting Approach Aimed at Sustainable Agriculture

**DOI:** 10.1021/acs.jafc.4c08328

**Published:** 2025-01-02

**Authors:** Gustavo Vinícios Munhoz-Garcia, Vanessa Takeshita, Jhones Luiz de Oliveira, Bruno Dalla Vecchia, Daniel Nalin, Camila de Werk Pinácio, Ana Laura Camachos de Oliveira, Brian Cintra Cardoso, Valdemar Luiz Tornisielo, Leonardo Fernandes Fraceto

**Affiliations:** † Center of Nuclear Energy in Agriculture, University of São Paulo, Av. Centenário 303, 13400-970 Piracicaba, SP, Brazil; ‡ Institute of Science and Technology, Sao Paulo State University, Av. Três de Março, 511 - Alto da Boa Vista, 18087-180 Sorocaba, SP, Brazil; § Superior School of Agriculture “Luiz de Queiroz”, University of São Paulo, Av. Pádua Dias, 11, 13418-900 Piracicaba, SP, Brazil

**Keywords:** nanoherbicide, zein, lignin, weed control, sustainability

## Abstract

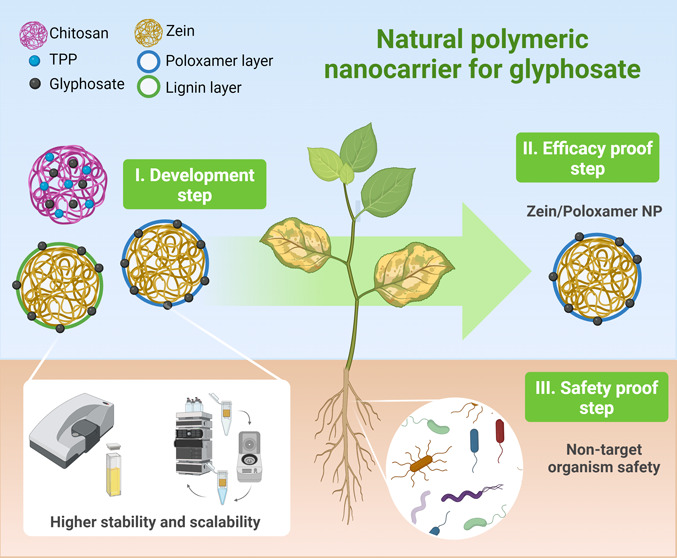

Polymer-based herbicide nanocarriers have shown potential for increasing the herbicide efficacy and environmental safety. This study aimed to develop, characterize, and evaluate toxicity to target and nontarget organisms of natural-based polymeric nanosystems for glyphosate. Polymers such as chitosan (CS), zein (ZN), and lignin (LG) were used in the synthesis. Nanosystem size, surface charge, polydispersity index, encapsulation efficiency, toxicity to weed species (*Amaranthus hybridus*, *Ipomoea grandifolia*, and *Eleusine indica*), and Roundup Ready (RR) crops, soil respiration, and enzyme activity were evaluated. The most stable system was the combination of ZN with the cross-linker poloxamer (PL), with higher weed control efficacy (90–96%) for *A. hybridus*, compared to commercial glyphosate (40%). No improvement was observed for *I. grandifolia* and *E. indica*. No glyphosate toxicity was observed in RR crops, soil respiration, or soil enzymes, indicating no toxic effects of the nanoformulation in these models. ZN-PL systems can be a promising alternative for glyphosate delivery, using environmentally friendly materials, with improved efficiency for weed control in agriculture.

## 1 Introduction

Glyphosate (C_3_H_8_NO_5_P, *n*-phosphomethyl glycine) is a nonselective herbicide that acts as an inhibitor of the enzyme EPSPS (5-enolpyruvylshikimate-3-phosphate synthase), preventing the synthesis of phenylalanine, tyrosine, and tryptophan.^1^ It has high water solubility (Sw 100 g L^–1^ at 20 °C), low affinity for lipophilic compounds (log Kow −3.2), low volatility (VP 0.0131 mPa),^2^ and four dissociation constants (p*K*_a_ ∼ 0.8, 2.6, 5.6, and 10.6).^2,3^ This herbicide plays a significant role in agriculture worldwide. Until the late 1980s, it was restricted to desiccation operations because it was not selective for cultivated plants and was expensive for farmers.^4,5^ However, since the approval of transgenic (RR) soybean with tolerance to glyphosate (cp4-epsps gene) and the reduction in the herbicide cost, its use in weed management has become versatile, being applied in more than one application during the crop cycle and increasing crop productivity.^6−8^ Developing other glyphosate-tolerant crops (such as RR cotton, corn, and sugar cane) and the exacerbated use of the herbicide in weed management operations has reduced its effectiveness due to the selection of resistant and tolerant weed biotypes.^9,10^

Despite this, glyphosate is still an essential herbicide in agriculture and plays a significant role in several cropping systems worldwide.^11^ Approximately 43–45% of herbicide applications in glyphosate-tolerant crops are made with glyphosate.^12^ In the U.S., glyphosate accounts for 31% (by volume) of herbicide applications in corn, 45% in soybeans, and 49% in cotton.^13^ Converted to the quantity of glyphosate, this represents 39,820, 33,790, and 5783 t, respectively. In Europe, glyphosate has recently been banned, but until 2022, it represented 30–50% of herbicides applied to perennial and annual crops.^14^ According to the Brazilian Institute for the Environment and Renewable Natural Resources (IBAMA), 266,088 t of glyphosate were marketed in Brazil in 2022.^15^ Countries like India use 600–700 tons in 77% of agricultural fields.^16,17^ In this sense, the absence of glyphosate in the crop system can lead to loss of weed control efficacy and an increase in the cost of agricultural production, with a 10–13% reduction in planted area.^5,14,18^ Given the reliance on this herbicide in current cropping systems and its continuous use over many years,^5^ a goal of modern agriculture is to maintain or increase the efficacy of glyphosate as a way to optimize sustainable weed management.

Exploring new technologies in the field is necessary for increasing sustainability in food production.^19^ Associating nanotechnology and agrochemicals can contribute to innovations in the safe use of herbicide products.^20^ According to Kah et al.,^21^ this is an effective and sustainable alternative, with great potential for application in developing new herbicide formulations, mainly because nanosystems can protect the active ingredient and increase the amount of the herbicide that reaches the target.^22^ Nanosystems also allow a reduction in the dose of the active ingredients applied, through increased efficacy,^23−25^ leading to more sustainable agriculture, as proposed by the FAO in the sustainable development goals (SDGs). For glyphosate, based on the large volume and frequency of applications, new sustainable formulations based on nanocarriers could aid its more efficient and safer (nontarget organisms and environment) use.

Natural-based polymers, like chitosan, zein, and lignin, are known as low-toxic, environmentally friendly, and biodegradable materials, and have been shown to be viable alternatives for nanoherbicide development.^20,26−28^ When used in nanoherbicide formulation, they can replace toxic components, like adjuvants and surfactants, promoting a greener formulation, to deliver more efficient herbicides.^28−30^ Few approaches considering biopolymers for synthesizing nanocarriers have been explored for glyphosate delivery.^31,32^

The research presented in Table 1 clarifies the current knowledge concerning nanosystems as glyphosate carriers. Jiang et al.^32^ used green materials to produce nanoemulsion-loaded glyphosate, in which the effective dose to control 50% of *Eleusine indica* individuals only reduced from 0.48 to 0.40 kg of acid equivalent (a.e.) ha^–1^. Chi et al.^33^ used attapulgite + poly(vinyl alcohol) to synthesize a temperature-controlled release of glyphosate; however, no improvement in weed control efficiency was observed. A polymeric nanosystem based on chitosan was developed by Rychter^34^ and tested against *Galinsoga parviflora*, *Rumex acetosa*, and *Chenopodium album*, although its efficacy in applicable field rates needed to be detailed. Recently, porous calcium carbonate microsphere-loaded glyphosate was developed by Zeng et al.,^35^ reducing glyphosate loss in plant leaves; however, no improvement in weed control was observed. These results point to the need to study nanotechnology as a tool for glyphosate delivery from an agronomic perspective, seeking alternatives to solve real agricultural problems.

**Table 1:** Number of Articles Related to Glyphosate and Nanotechnology Available on the Web of Science Database Based on the Searched Term

	results^a^		
research terms	general^b^	applied^c^	main study areas
glyphosate + nanoparticles	324	9	analytical chemistry (26.8%), environmental sciences (19.1%), chemistry multidisciplinary (12.9%), materials science (12%), and nanoscience nanotechnology (11.4%)
glyphosate + delivery system	46	7	agronomy (30.4%), entomology (23.9%), and environmental sciences (17.4%)
glyphosate + encapsulation	8	1	materials science (28.5%) and nanoscience nanotechnology (28.5%)
glyphosate + loading + polymers	13	1	analytical chemistry (38.4%) and agronomy (15.8%)
nanoparticle + glyphosate + encapsulation	0		
glyphosate + encapsulation + polymers	0		

a Duplicated articles were not removed within the research terms.

b General results were considered for all of the articles returned in the search.

c Applied results were considered those that used nanomaterials as a delivery system for glyphosate.

Rather than nanosystem composition, the synthesis process is also important in nanosystem development,^36^ considering that after the proof of concept, the technology may become available for scale-up, followed by field tests, registration, production, and commercialization.^37^ The translation of nanoformulation production from the laboratory to large scale is challenging due to the variation in reproducibility of nanosystem properties (size, shape, loading) and the complexity of the synthesis steps.^38^ Simple and reproducible synthesis steps can aid the nanoformulation scale-up using fully automated tools.^39^ Considering the need for new technologies to control weeds with a sustainable approach, the role of green nanobased formulations as herbicide carriers is highlighted to provide a possible and reliable class of herbicides to the market.

The wide use of glyphosate in weed management, the impacts of herbicides on the environment, and the sustainable goals for modern agriculture should direct agricultural research in the coming years. Developing technologies that increase the effectiveness of herbicides and reduce their environmental impact is necessary to maintain the sustainability of the agroecosystem. The current study aimed to design and characterize the nanoformulation aspects (stability, size, shape, charge, and dispersion) of natural-based polymeric nanosystems for delivery of glyphosate to plants. We also evaluated the toxicity to target weed species (*A. hybridus*, *Ipomoea grandifolia*, and *E. indica*,), nontarget plants (RR soybean and cotton), and soil microorganisms. We hypothesized that (I) it is possible to use natural polymers as glyphosate carriers in a two-step synthesis; (II) polymeric constitution can change the nanosystem characteristics and efficacy; (III) weed species show different tolerance to the nanosystem; (IV) glyphosate polymeric nanosystems can affect RR crops; and (V) nanosystems can influence soil enzyme activity. This work also points out an exploratory approach in nanoparticle development using natural-based polymers and shows the effect of weed species on the nanosystem efficacy.

## 2 Materials and Methods

### 2.1 Materials

Chitosan, zein, tripolyphosphate, lignin, poloxamer (Kolliphor PS 80), 4-methylumbelliferyl β-d-glucopyranoside (MUB-G), 4-methylumbelliferyl sulfate potassium salt (MUB-S), acetone, and ethanol were purchased from Sigma-Aldrich (Sigma-Aldrich, Chem. Co., San Loius, MO). ^14^C-Glucose (radiochemistry purity >95%) was purchased from American Radiolabeled Chemicals (Inc., St. Louis, MO). Seeds of *A. hybridus*, *E. indica*, and *I. grandifolia* were purchased from Agrocosmos (Cosmos Agrícola Produção e Serviços Rurais Ltd. SP, Brazil). Glyphosate (isopropylamine salt, 840 g kg^–1^ acid equivalent, a.e.), RoundUp (360 g a.e. L^–1^), and seeds of *Glycine max* and *Gossypium hirsutum* tolerant to glyphosate (RR) were purchased from local commerce.

### 2.2 Nanoformulation Design–Exploratory Approach

An exploratory approach was used to find possible nanosystems for glyphosate encapsulation in polymeric nanostructures. Chitosan (CS) was used alone and in combination with tripolyphosphate (TPP), and zein (ZN) was combined with poloxamer (PL) or lignin (LG) for preliminary testing as glyphosate carriers. Initially, the nanosystems were selected when the formulation presented a hydrodynamic size <1000 nm and polydispersity index (PDI) <0.5. After nanoparticle formation, encapsulation efficiency (EE) >40% was considered an eliminatory criterion for nanosystem development.

#### 2.2.1 Chitosan/Tripolyphosphate Nanoparticles

CS/TPP nanosystems were prepared according to Calvo et al.^40^ with modifications. Initially, aqueous solutions with 0.1, 0.3, and 0.5% (m/v) of chitosan at pH 4.5 were prepared by the addition of 100, 300, and 500 mg of chitosan in distilled water (0.2% of acetic acid) under magnetic stirring for 12 h at room temperature. The solution was filtered in a syringe filter (0.45 μm Millipore) and kept in the dark. Tripolyphosphate solutions were prepared at 0.1, 0.08, and 0.05% (m/v) by diluting 100, 80, and 50 mg of tripolyphosphate in distilled water. First, we tested the influence of glyphosate addition in CS 0.1% or TPP 0.1% solution. To test the effect of CS concentration in nanoparticle development, CS solutions (0.1, 0.3, and 0.5%) were tested with TPP at 0.1%. To test the influence of TPP on nanoparticle formation, CS 0.1% was used combined with TPP solutions (0.1, 0.08, 0.05, and 0.01%). In all treatments, 6 mL of TPP solution was added to 10 mL of solution of CS containing 24 mg of glyphosate under magnetic stirring for 20 min. The final concentration was 1.5 mg mL^–1^.

#### 2.2.2 Zein/Poloxamer Nanoparticles

A hydroethanolic solution with 2% (m/v) of zein was prepared by adding 2 g of zein to 100 mL of ethanol/water (85:15, v/v) under magnetic stirring and kept overnight at room temperature, as per de Oliveira et al.^41^ After zein dilution, the solution was submitted to a thermal bath at 75 °C for 5 min, before being centrifuged for 25 min at 4000 rpm and filtered in a 0.45 μm syringe filter (Millipore), and the pH was adjusted to 4.5 with HCl (1 M). A 2% (m/v) poloxamer solution was prepared by diluting 2 g of the commercial poloxamer in 100 mL of distilled water under agitation. Then, 10 mL of ZN solution was mixed with 30 mL of PL solution containing different concentrations of glyphosate (60, 90, and 120 mg mL^–1^) under magnetic stirring for 20 min. The solutions were concentrated to 30 mL in a rotary evaporator at 45 °C. The glyphosate concentrations were 2, 3, and 4 mg mL^–1^ in the final solutions.

#### 2.2.3 Zein/Lignin Nanoparticles

The zein solution (2%, m/v) was prepared as described above^41^ and a 1% (m/v) lignin solution was prepared by adding 1 g of lignin to distilled water and stirring. Then, 60 and 90 mg of glyphosate were diluted in LG solution at room temperature, and 10 mL of ZN solution was added to the solution and kept for 20 min under magnetic stirring. The solutions were concentrated to 30 mL as described above. The final concentration of glyphosate was 2 mg mL^–1^, since the higher concentrations precipitated.

### 2.3 Nanoformulation Characterization

After the exploratory step, the selected nanosystems were developed and the nanoparticle characteristics were analyzed when the formulations were reproduced. Formulations based on CS/TPP, ZN/PL, and ZN/LG were prepared as described above (see Section 2.2). The nanoparticles were characterized by hydrodynamic size, surface charge, polydispersity index (PDI), and encapsulation efficiency initially and 60 days after the preparation. To determine the hydrodynamic size, surface charge, and PDI, the nanoformulation was diluted 1000 times in distilled water, and 1 mL of the diluted solution was submitted to a ZetaSizer Nano ZS90 (Malvern Instruments, U.K.) at a fixed angle (90°) at 25 °C, in three replicates.

Liquid chromatography–mass spectrometry (LC-MS/MS) determined the encapsulation efficiency.^42^ The mobile phase was 95% water containing ammonium formate (50 mM) and 5% acetonitrile in an isocratic mode at 0.35 mL min^–1^. The stationary phase consisted of a HiliCpak collum (2 mm × 150 mm, 5 μm) operated at 40 °C. The injection volume was 25 μL. The source parameters were nitrogen gas at 140 °C and a flow of 12 L min^–1^, nebulizer pressure at 30 psi, and capillarity voltage at 3 kV. The equipment was operated in negative electron spray ionization (ESI-), the precursor and product ions were 168 > 150 and 168 > 63, respectively, with fragmentation energy of 135 V and collision energy of 8 V. Seven glyphosate concentrations (50, 75, 100, 250, 500, 1000, and 2000 ng mL^–1^) were prepared and three replicates were injected into LC-MS/MS to construct the analytical curve (Figure S1).

The encapsulation efficiency was measured by adding 400 μL of each formulation in cellulose ultrafilters (Microcon 10 kDa, Millipore), and then they were centrifuged (Hitachi CF16RXII, Hitachi Koki Co., Ltd., Indaiatuba, SP, Brazil) under 4500 rpm, for 10 min, at 20 °C. 25 μL of the filtered solution was diluted 1000 times in ultrapure water, and three replicates were injected into LC-MS/MS. The amount of nonencapsulated glyphosate was determined from the total in nonfiltered solution (eq I).
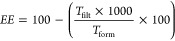
Iwhere EE is the total of glyphosate encapsulated (%), *T*_form_ is the total of glyphosate in the initial formulation (mg), and *T*_filt_ is the total of glyphosate in the filtered solution (mg). The nanosystems were selected based on size, surface charge, PDI, EE, and repeatability.

The shape and size of the most interesting NPs were investigated by atomic force microscopy (AFM). Sample preparation was performed by diluting 1 μL of nanoparticle suspension in ultrapure water (1:100000, v/v), followed by deposition onto a silicon plate and drying in a desiccator. The data were obtained using an Easy Scan 2 instrument (Basic AFM- Pattern BT02217; Nanosurf, Switzerland) operated in noncontact mode and equipped with a TapAl-G cantilever (BudgetSensor, Bulgaria) at a scan rate of 90 Hz. Images were processed by using Gwyddion software. The size counts were fitted in a normal distribution.

### 2.4 Initial Biological Activity

Four nanoformulations were submitted to a biological assay to determine their effects on *A. hybridus*. The experimental design was completely randomized, with 10 treatments and six replicates. The treatments consisted of RoundUp (Glyphosate commercial formulation at rate of 720 g a.e. ha^–1^), ZLF2 (ZN/LG nanosystem at 2 mg a.i. mL^–1^), ZPF1 (ZN/PL nanosystem at 2 mg a.i. mL^–1^), ZPF2 (ZN/PL nanosystem at 3 mg a.i. mL^–1^), and ZPF3 (ZN/PL nanosystem at 4 mg a.i. mL^–1^) at rates of 720 and 360 g a.e. ha^–1^, and a control group (without herbicide application). The experimental units consisted of 300 cm^3^ pots filled with soil/substrate mixture (1:2, m/m) and an *A. hybridus* plant with 6–8 fully expanded leaves. The plant species were selected based on their high sensitivity to glyphosate, according to the results of a dose–response curve assay (data not shown), where *A. hybridus* was the most sensitive species, *E. indica* presented middle sensitivity, and *I. grandifolia* presented low sensitivity to glyphosate.

The technical-grade glyphosate (84% of glyphosate) was considered in the nanosystem constitution. The application solutions were prepared according to Table S1 (Supporting Information), diluting the respective amount of each formulation in deionized water (pH 6.5). The glyphosate rate (a.e. ha^–1^) was calculated considering the pot area (3.84 × 10^–3^ m^2^). Each experimental unit received 1 mL of solution and was applied with manual spray. After herbicide application, the plants were kept in a growing chamber with a controlled environment (21–27 °C, 12 h photoperiod, and 60% air humidity), with daily irrigation directly to the soil surface. The control efficacy evaluation was performed 7, 14, and 21 days after herbicide application (DAA) using the visual damage scale. At 21 DAA, the plants were removed from the pots and the fresh weight was measured. The nanoformulation was selected based on the efficacy in controlling the weed plants. Considering industrial and agronomic perspectives, systems with higher loading capacity (the amount of glyphosate in the solution) were prioritized.

### 2.5 Toxicity to Weed Species

The zein/poloxamer nanosystem, or ZPF3, was selected as the model platform for the glyphosate carrier (based on results from Section 2.2 assays), and its efficacy was tested against *I. grandifolia* and *E. indica* plants. The plants were grown in a growing chamber under the conditions described above (Section 2.3). The experimental units consisted of pots filled with soil/substrate mixture (1:2, m/m) and 1 plant per pot. The treatments consisted of RoundUp (Glyphosate commercial formulation at rate of 720 g a.e. ha^–1^), nanoformulation ZPF3 at rates of 720 and 360 g a.e. ha^–1^, and a control group (without herbicide application), with six replicates. The application solution was prepared according to the respective formulation, considering the pot area described in Section 2.3, Table S2. The application was performed with a manual spray, as described above. The control efficacy evaluation was performed at 7, 14, and 21 DAA using a visual damage scale, for *I. grandifolia* and *E. indica*, and at 21 DAA, these plants were removed from the pots, and the fresh weight was measured.

### 2.6 Selectivity to Tolerant Crops

Nanosystem toxicity to RR crops (*G. max* and *G. hirsutum*) was tested in an entirely randomized assay with six repetitions. The experimental design consisted of two plant species (soybean and cotton), two glyphosate formulations (ZPF3 and RoundUp at a rate of 720 g a.e. ha^–1^), and a control group without herbicide application. Three soybean and cotton seeds were sown in pots (300 cm^3^) filled with a soil/substrate mixture and grown in a growing chamber with controlled environmental conditions (see Section 2.4 for more details). For solution preparation, 5.38 μL of RoundUp was mixed with 7 mL of distilled water, and 584 μL of ZPF3 was diluted in 6.41 mL of distilled water. One plant was grown per pot until 25 days after emergence (DAE), and 1 mL of each formulation was applied per pot with a manual spray, as described above. The toxicity was evaluated according to visual injuries at 7, 14, and 21 DAA. At the end of the experiment, the plants were removed from pots to obtain fresh weight.

### 2.7 Soil Respiration Assay

A respirometry study was conducted to evaluate the formulation effects on soil respiration,^43^ and an enzyme activity assay was carried out to understand its effects on β-glucosidase and arylsulfatase activity in the soil.^44^ The experimental design was two glyphosate formulations (ZPF3 and RoundUp) applied at 1440 g a.e. ha^–1^ (2 times the recommended dose and the standard dose used in the field) and a control treatment without herbicide, with three replicates. The soil was an Ultisol, collected in an area covered with *Brachiaria spp.* at a depth of 0–20 cm. The collected soil was sieved at 2 mm and kept at room temperature for 1 week prior to the experiment implantation, until field capacity and humidity tests were finished. The soil field capacity was 32%, and humidity was 12.2%. The soil field capacity was kept at 75% during the experiment.

For respirometry studies, 10 g of soil was weighed (considering soil humidity) and accommodated in a biometric flask with a CO_2_ trap (10 mL of NaOH 0.2M) at the side handle.^43^ The experimental units were kept in the dark at 25 °C and evaluated over time (0, 7, 14, 21, and 28 DAA) using destructive samples. The glyphosate dose (6 μg) in the experimental units was calculated based on soil mass contained in 1 ha (0–20 cm depth). RoundUp solution was prepared by adding 43 μL of a stock solution of RoundUp (3.6 mg a.e. mL^–1^) in 11 mL of water, and for the ZPF3 solution, 29 μL of the formulation was applied in 11 mL of water. The volume of water was calculated to adjust the soil humidity to 75% of the soil field capacity and used as a vehicle for the formulations. In total, 688 μL of the solution containing RoundUp, ZPF3, or water were applied to the soil surface with a micropipette. A 100 mg mL^–1^ and 3.1 kBq mL^–1^ of ^14^C-glucose solution was prepared in distilled water and 500 μL was applied on the soil at 0, 7, 14, 21, and 28 days after herbicide application. The amount mineralized to ^14^CO_2_ was measured using a liquid scintillation spectrometer (LSS), at 48 h after ^14^C-glucose application in the soil, due to rapid mineralization. In each evaluation period, two 1000 μL aliquots from a NaOH solution were sampled from each experimental unit and added to a vial with 10 mL of Insta-Gel Plus scintillation solution. LSS quantified the radioactivity for 5 min. The amount of ^14^C-glucose converted to ^14^CO_2_ was calculated compared to the amount applied initially.

### 2.8 Enzyme Activity Assay

The enzyme activity assay was conducted in 0.2 cm^3^ plastic pots with 20 g of soil. The herbicide dose was calculated according to the soil mass. The experimental design consisted of an entirely randomized assay, with three treatments (Control group, RoundUp, and ZPF3, at a rate of 1440 g a.e. ha^–1^) and three repetitions, evaluated at 0, 7, 14, 21, and 28 days after herbicide application in soil, in destructive samples. The soil used was collected and later prepared with moisture adjusted to 75% by adding 1.8 mL of deionized water (in each experimental unit) before herbicide application in the same way as described in Section 2.6. A glyphosate dose of 12 μg a.e. was considered based on the soil field recommendation and soil mass used in the assay. The RoundUp solution was prepared by adding 54 μL of a stock solution of RoundUp (3.6 mg a.e. mL^–1^) in 32 mL of water, and for the ZPF3 solution, 58 μL of the formulation was applied in 32 mL of water. Subsequently, 2 mL of the work solution was applied to each experimental unit with an automatic micropipette, then the pots were covered with a perforated plastic film to reduce water loss. Soil moisture was kept at 75% of soil field capacity during the experiment, and deionized water was added by mass difference when needed.

The enzymes evaluated were β-glucosidase and arylsulfatase, using a fluorescent-based method with modifications.^44,45^ A calibration curve using 4-Methylumbelliferone (MUB) (0.01–2 μmol mL^–1^) in a fluorescence reader (Microplates Tecan Infinite 200 Pro) was performed in soil extract. The fluorescence was excited at 365 nm, and the emission was measured at 460 nm. The quantification limit of MUB was considered the intercept (*b*) of the linear regression (*y* = *ax* + *b*), and the equation obtained was *y* = 4000000*x* + 5298 (*r*^2^ = 0.999, *p* < 0.05), where *y* is the fluorescence emitted by MUB and *x* is the MUB concentration in the sample.

Fluorescent probes linked to β-glucosidase (4-methylumbelliferyl β-d-glucopyranoside, MUB-G) and arylsulfatase (4-methylumbelliferyl sulfate potassium salt, MUB-S) were used to determine enzyme activity in the soil. At each evaluation time, the soil was homogenized, two soil aliquots of 500 mg were removed from each pot and dried at room temperature, and the enzymes were extracted from the soil. The extraction procedure was performed by adding 25 mL of a 50 mM sodium acetate buffer solution (pH 6, equal to the soil samples) under orbital stirring for 30 min at 200 rpm. The samples were then centrifuged (Hitachi CF16RXII, Hitachi Koki Co., Ltd., Indaiatuba, SP, Brazil) for 5 min at 4500 rpm at room temperature. Two aliquots of 1 mL of soil extract were mixed with 425 μL of 4 mg mL^–1^ solution of MUB-G (0.8 μmol) or 185 μL of 4 mg mL^–1^ solution of MUB-S (0.25 μmol mL) in a plastic microtube (2 mL), incubated for 24 h in a thermal bath at 37 °C. Subsequently, four 200 μL aliquots of each sample were placed in a dark microplate for fluorescence reading. The fluorescence emission results were transformed to enzyme activity (μmol MUB g^–1^ day^–1^) using the above equation (y = 4000000*x* + 5298), considering the soil mass and total solution volume.

### 2.9 Statistical Analysis

The data from the experiments were submitted to normality, homogeneity, and homoscedasticity tests. When the variance presented normal and homogeneous distribution, an analysis of variance (ANOVA) was performed to identify the treatment effect and Tukey’s HSD test to compare the means. When assumptions were not met, the data were transformed by using the Yeo-Johnson transformation. A cubic model was adjusted for the respiration data over the incubation time. The significance of 5% (*p* < 0.05) was considered in all statistical tests. The graphs and analysis were elaborated using Origin 2024 software (Version 10.100178, OriginLab Corporation, Northampton, MA).

## 3 Results and Discussion

### 3.1 Nanoformulation Design and Properties

The main results for nanoparticles based on glyphosate and polymer combinations are presented in Table 2. CS combined with TPP at concentrations of 0.05 to 0.1% resulted in nanoparticle formation (with sizes less than 1000 nm, PDI < 0.5) (Table 2 and Figure S2). Increasing the CS concentration prevented nanoparticle formation (Figure S3), and diluting glyphosate in CS or TPP solution did not influence the nanosystem development. The ZN combinations with LG and PL led to nanoparticle formation (Table 2, Figures S4 and S5). The increase in the glyphosate concentration from 2 to 4 mg mL^–1^ led to instability in the LG + ZN system but not in the ZN + PL system (Table 2). Some studies have found these polymers to be herbicide carriers.^20,46−51^ However, few studies have been performed on nanoformulation for glyphosate delivery,^31,46,52^ leading to little knowledge and exploration of how nanoparticles can affect glyphosate efficacy. In addition, previous works used different types of materials (polymers, oils, metals) combined in various and complex synthesis steps in the nanosystem design, differing from our work, where only the polymers and stabilizers were used in a two-step synthesis (I) glyphosate dilution in the aqueous phase + the polymer in another phase (aqueous or organic, depending on the system) and (II) solvent removal by rotary evaporation. In this sense, combinations using CS 0.1% + TPP 0.1%, ZN 2% + LG 1%, and ZN 2% + PL 2% were considered potential nanosystems and were selected for stability and repeatability studies.

**Table 2:** Nanosystem Design in an Exploratory Analysis Based on the Biopolymers Chitosan, Zein, and Lignin^a^

formulation	glyphosate addition	final concentration (mg a.i. mL^–1^)	nanoparticle formation	encapsulation efficiency (%)
CS 0.1% + TPP 0.1%	in CS solution	1.5	yes	within 40–60
CS 0.1% + TPP 0.1% (CTF)*	in TPP solution	1.5	yes	within 40–60
CS 0.3% + TPP 0.1%	in CS solution	1.5	no	not evaluated
CS 0.5% + TPP 0.1%	in CS solution	1.5	no	not evaluated
CS 0.1% + TPP 0.08%	in CS solution	1.5	yes	within 40–60
CS 0.1% + TPP 0.05%	in CS solution	1.5	yes	within 40–60
CS 0.1% + TPP 0.01%	in CS solution	1.5	yes	<40
LG 1% + ZN 2% (ZLF2)*	in LG solution	2	yes	within 40–60
LG 1% + ZN 2%	in LG solution	3	no	not evaluated
LG 1% + ZN 2%	in LG solution	4	no	not evaluated
PL 2% + ZN 2% (ZPF1)*	in PL solution	2	yes	within 40–60
PL 2% + ZN 2% (ZPF2)*	in PL solution	3	yes	within 40–60
PL 2% + ZN 2% (ZPF3)*	in PL solution	4	yes	within 40–60

a Asterisks (*) represent the formulations selected for further studies.

### 3.2 Nanoformulation Characterization

**Figure 1:**
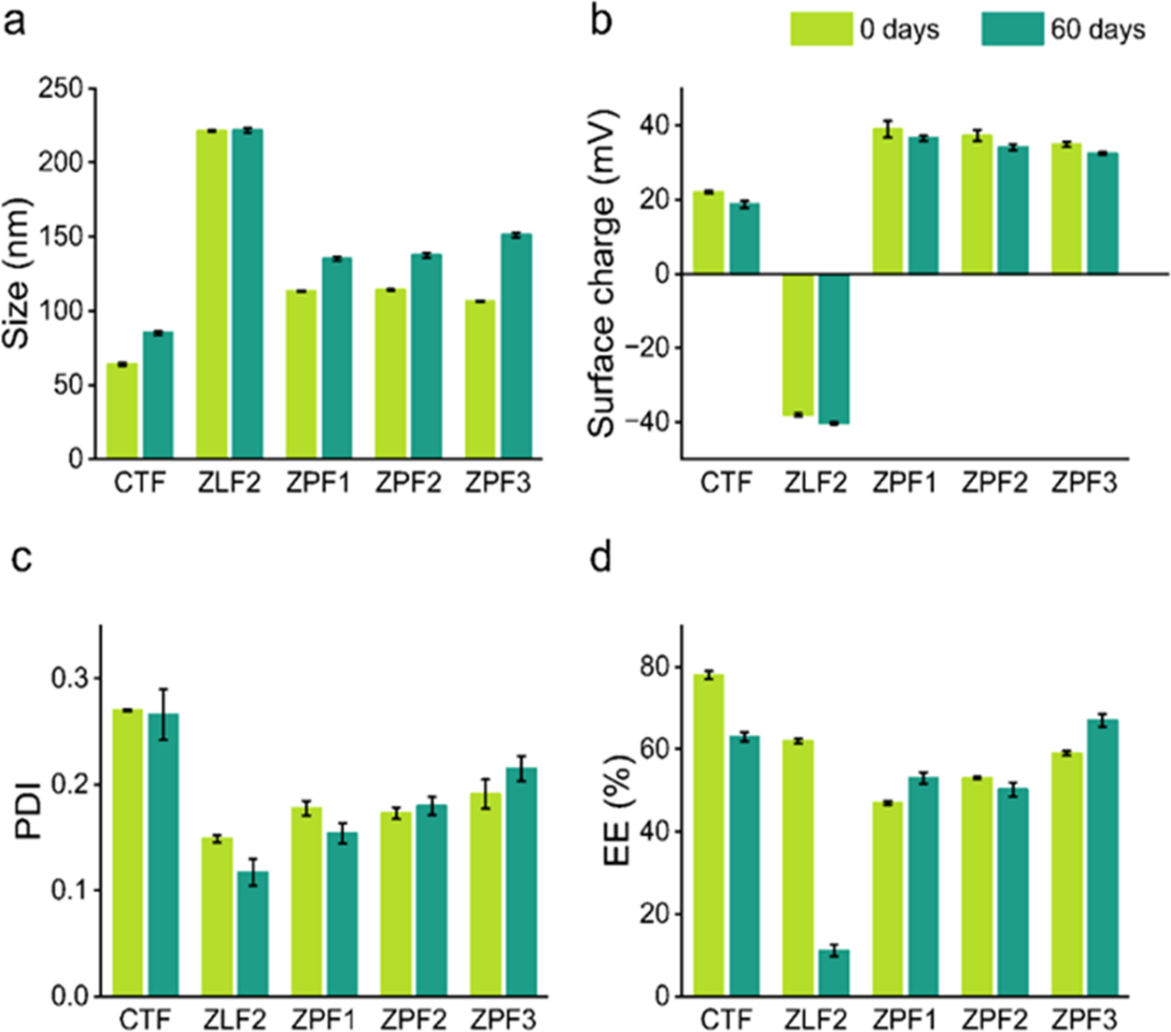
Nanosystem stability in relation to size (a), surface charge (b), dispersion (PDI, (c)), and encapsulation efficiency (EE, (d)) of selected glyphosate carriers. 0 Days represents the analysis on the day of nanoformulation synthesis, and 60 days represents storage in the dark, at 21–25 °C for 60 days after synthesis. CTF—chitosan/TPP, ZLF2—zein/lignin, ZPF1—zein/poloxamer 2 mg mL^–1^, ZPF2—zein/poloxamer 3 mg mL^–1^, ZPF3—zein/poloxamer 4 mg mL^–1^.

The size, surface charge, PDI, and encapsulation efficiency of the selected nanosystems are listed in Figure 1a–d. A few changes occurred during nanosystem storage. Initially (0 days), the system based on CS/TPP (CTF) presented a size of 64 ± 1 nm, positive charge of 22 ± 2.4 mV, PDI of 0.27, and EE of 78 ± 3.2%. After 60 days, the size measured was 85 ± 1.1 nm, charge of 19 ± 0.9 mV, PDI of 0.27, and EE of 63 ± 0.2%, resulting in a loss of glyphosate from the nanoparticle over time. CS-based nanosystems are positively charged, nontoxic, and green alternatives to agrochemical formulations.^53^

Glyphosate has two groups ionized at CS in pH 4.5, which are negatively charged. This makes the interaction with CS possible; however, using negatively charged cross-linkers such as TPP is essential to promote nanoparticle formation,^54^ since the reduction in TPP concentration prevented encapsulation (Table 2 and Figure S2). Electrostatic interaction is responsible for particle formation in chitosan/TPP systems.^51^ In this work, the cationic chitosan and anionic forms (glyphosate and TPP) in solution formed the nanosystem. Given the positive ζ-potential and the mechanisms of NP formation for nanosystems based on chitosan/TPP^55^ observed for other acid herbicides,^50^ it is likely that both glyphosate and TPP act as cross-linkers and are located under the chitosan molecules, forming a wide range of small particles carrying glyphosate.

Initially, the system based on LG/ZN (ZLF2) presented a size of 226 ± 0.6 nm, a negative charge of −38 ± 0.7 mV, a PDI of 0.15, and an EE of 63 ± 13.2%. The size (222 ± 1.4 nm), charge (−40 ± 0.3 mV), and PDI (0.11) of ZLF2 presented a low variation after storage. However, the EE was reduced to 11.1%. This system allows for NP formation by associating negatively charged glyphosate with positively charged zein molecules, forming a complex of associated zein-glyphosate with lignin on the outside due to a negatively charged particle (Figure 1). The negative charge on the outside of NPs is due to LG surface phenolic hydroxyl groups responsible for the electrostatic repulsion.^56^ Furthermore, LG-based systems are known for their excellent stability in solution.^57^ However, in the research presented here, the interactions driving the system were weak, leading to a reduced level of glyphosate encapsulation over time.

The systems based on ZN/PL (ZPF1, ZPF2, and ZPF3) presented similar characteristics, with a size ranging from 106 to 113 nm, positive charge from 35 to 39 mV, PDI from 0.17 to 0.19, and EE from 47 to 59%. After 60 days, the systems presented similar sizes, PDI, positive charge, and EE, resulting in a stable system (Figure 1a–d). Similar results of size (121–136 nm), charge (17–23 mV), and PDI (0.15–0.25) were found for the ZN/PL system as rutin carriers.^58^ As an atrazine carrier, the ZN/PL nanoparticle presented a size of 130–170 nm, positive charge of 12 mV, PDI < 0.25, and EE of 90%.^20^

Zein is a highly versatile protein for encapsulating hydrophilic^59^ and hydrophobic^60^ compounds. The specific mechanisms of interaction will depend on the compound in question. In our systems with glyphosate, we hypothesize that ZN/PL has a hydrophobic nucleus due to the compact organization of the zein structure.^61^ The hydrophilic interface formed by zein/poloxamer allows for the location of glyphosate by its hydrophilic characteristics.^2^ This is similar to the association of ZN/PL NPs with ionic compounds presented by El-Lakany and colleagues.^62^ However, given that the ζ-potential is positive, it can be inferred that there must be cationic chains of zein in a relaxed state outside of the NPs.

**Figure 2:**
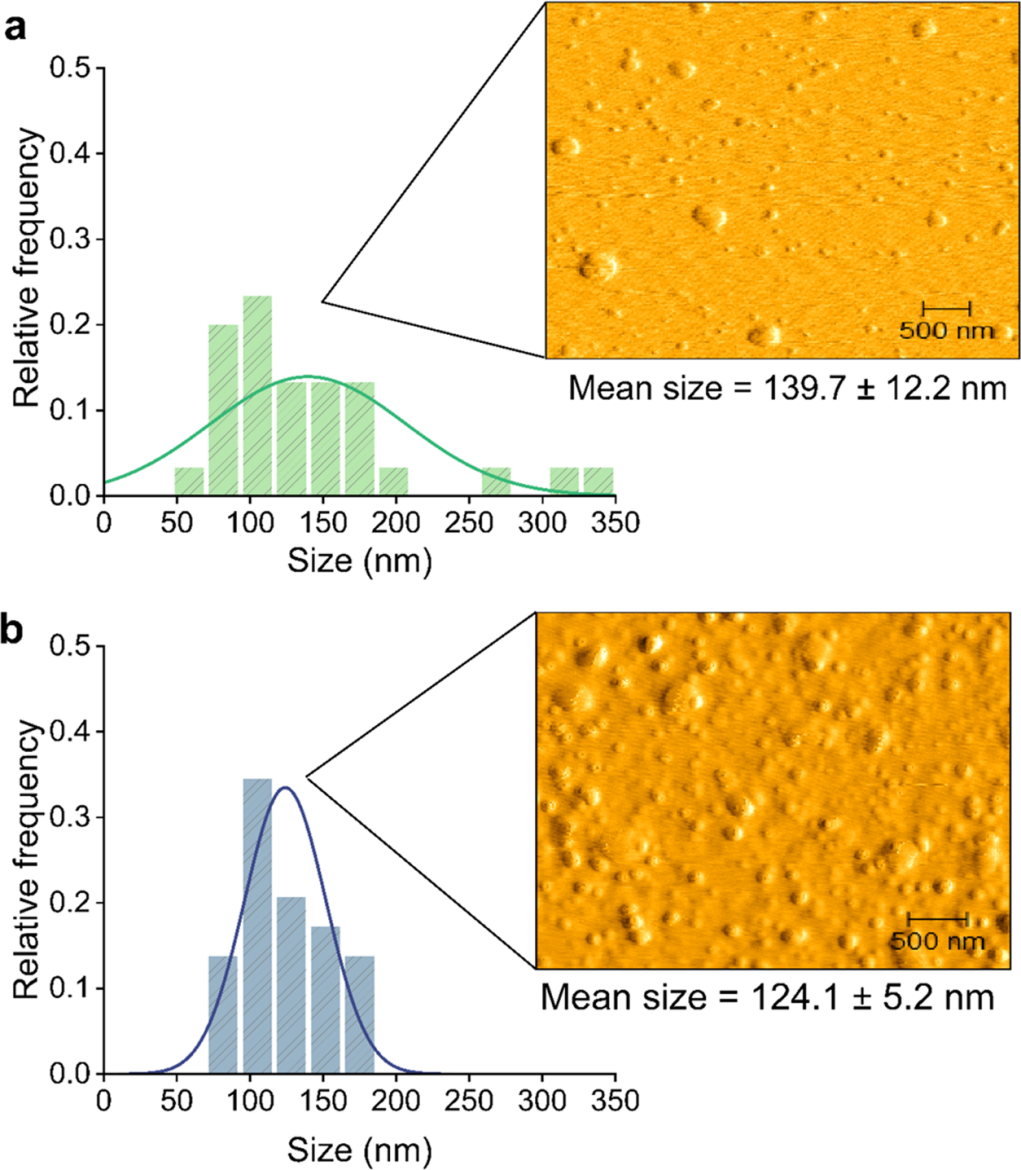
Particle shape and size distribution (measured by AFM) of ZPF1 (a) and ZPF3 (b). The values represent the mean ± the standard error (*n* = 30). ZPF1—zein/poloxamer 2 mg mL^–1^, ZPF3—zein/poloxamer 4 mg mL^–1^.

Measurements of size, charge, and PDI were performed in different batches of CTF, ZLF, and ZPF formulations, and the results showed similar parameters within the batches, indicating good reproducibility of the formulations (Figure S6). The findings indicate the potential use of ZN/PL systems as herbicide carriers and suggest the possibility of developing a nanoformulation for glyphosate delivery to plants. Based on the efficacy results (presented in the following sections), the most interesting systems were ZPF1 and ZPF3, which showed a spherical shape when diluted in water, as measured by AFM, and a size distribution range similar to that observed by DLS analysis (Figure 2). Overall, the design step demonstrates the effect of polymers and cross-linkers on nanosystem formation and stability, enhancing knowledge about polymeric nanoparticle-based herbicide carriers.

### 3.3 Initial Biological Activity

**Figure 3:**
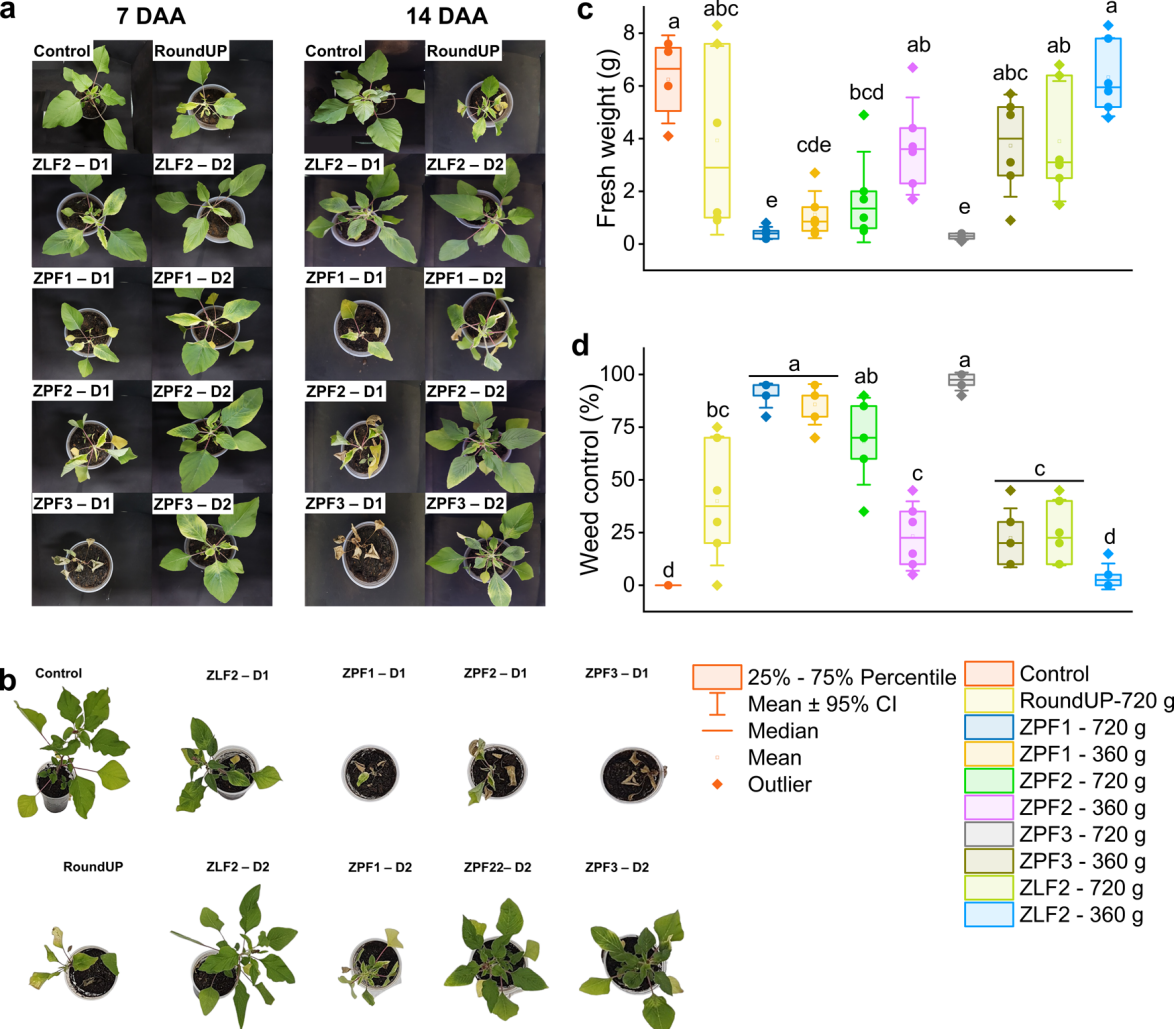
Symptom evolution at 7 and 14 DAA (a), 21 DAA (b), fresh weight (c), and weed control (d) of *A. hybridus* plants with different glyphosate formulations at 21 DAA. The rates used for nanoformulations were 720 g a.e. ha^–1^(D1) and 360 a.e. ha^–1^(D2). Boxes with the same lowercase letters did not differ according to Tukey’s test (*p* < 0.05).

The evolution of glyphosate symptoms in *A. hybridus* plants from 7 to 14 DAA was evaluated to identify if nanosystems promote faster plant damage, and the results are presented in Figure 3a. The formulations ZPF1, ZPF2, and ZPF3, at 720 g a.e. ha^–1^ presented faster plant injuries, and ZPF3 led to plant death at 7 DAA (Figure 3a). At 14 DAA, the symptoms of ZPF1 and ZPF2 evolved and were similar to those of commercial glyphosate (Figure 3a). At 21 DAA (Figure 3b), the formulations ZPF1 and ZPF3 (720 g a.e. ha^–1^) presented fresh weights 13 times less than the plants treated with commercial glyphosate and 24 times less than the control treatment without herbicide (Figure 3c). Meanwhile, commercial glyphosate reduced the fresh weight by only 1.9 times compared to that of the control treatment (Figure 3c). The nanosystem ZLF2 was not efficient in controlling the weed plants, with no differences in fresh weight reduction and weed control (25%) compared to glyphosate (40%) or to the control group (Figure 3d). This indicates that based on glyphosate concentration dependence, ZN/LG nanoparticles can reduce or interfere with herbicide efficacy in weed plants.

On the other hand, the control efficacy (Figure 3d) was higher for ZPF1 and ZPF3 (90–96%) at full dose (720 g a.e. ha^–1^), compared to commercial glyphosate (∼40%). When the dose was reduced to 360 g a.e. ha^–1^, ZPF1 presented a mass reduction of 6.7 times, similar to ZPF3 at full dose (720 g a.e. ha^–1^), with control efficacy of ∼85%, and no differences between ZPF1 and ZPF3 at full dose (Figure 3d). Glyphosate presents high toxicity for susceptible populations of *A. hybridus*, where 5–220 g a.e. ha^–1^ provided 50% control.^63−65^ Therefore, the possible changes in glyphosate efficacy caused by the nanosystem can be evaluated using this weed species, acting as an indicator of the nanosystem efficacy. The hypothesis-driven NP/plant interaction will be later discussed (Section 3.5)

The results of the current study generally indicate that nanoparticles improved glyphosate toxicity to *A. hybridus* plants, mainly when the glyphosate concentration was high (4 mg mL^–1^) in the nanosystem. However, the composition of nanoparticles can change this effect, playing an important role in the design step. From an agronomic perspective, the ZPF1 and ZPF3 systems could be suitable as glyphosate carriers. From an industrial perspective, the more loaded system (ZPF3) is easier to use in field experiments. In light of these findings, ZPF3 was selected for further experimental testing.

### 3.4 Efficacy in Weeds

**Figure 4:**
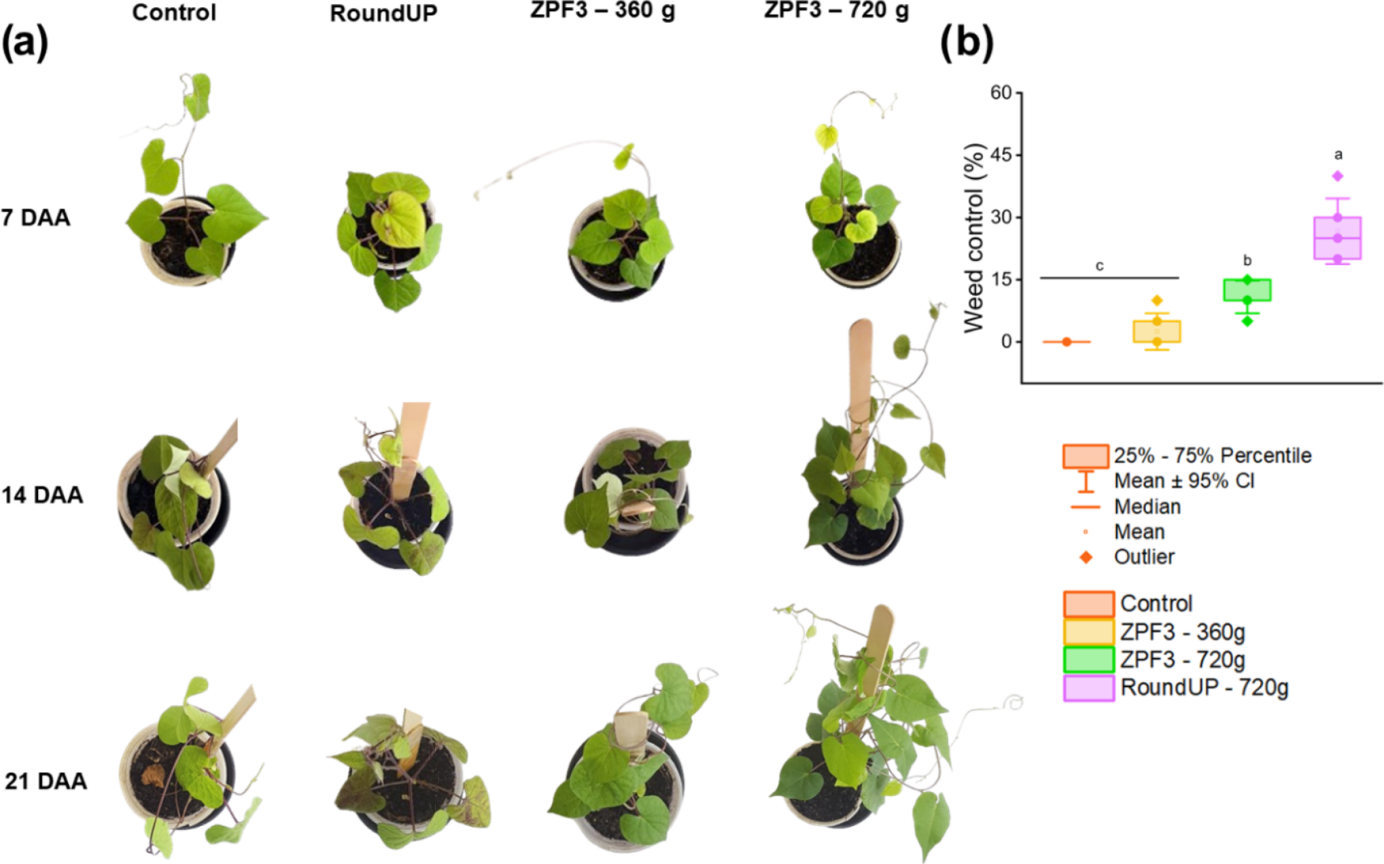
Symptom evolution (a) and weed control efficacy (b) of glyphosate formulations against *I. grandifolia* plants, at 21 DAA. Boxes with the same lowercase letters did not differ according to Tukey’s test (*p* < 0.05).

**Figure 5:**
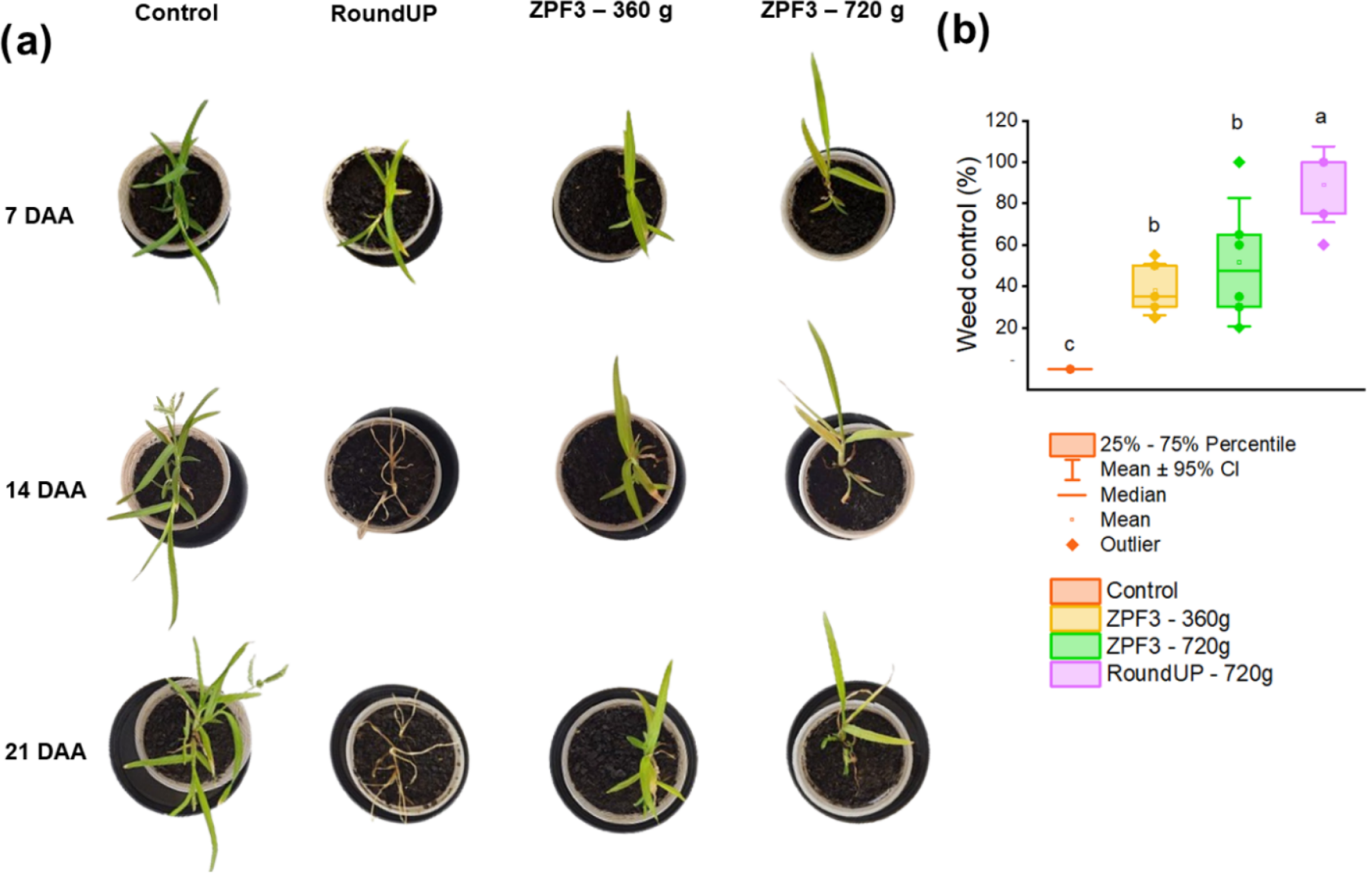
Symptom evolution (a) and weed control efficacy (b) of glyphosate formulations against *E. indica* plants, at 21 DAA. Boxes with the same lowercase letters did not differ according to Tukey’s test (*p* < 0.05).

Significant differences were found between the treatments for weed control of both species (*p* < 0.05), but no differences were found in the fresh weight reduction (*p* > 0.05). The weed control efficacy of glyphosate formulations and the evolution of symptoms in *I. grandifolia* and *E. indica* are presented in Figures 4a and 5a. In *I. grandifolia* plants, the commercial glyphosate (RoundUp) provided higher control efficacy (26.7 ± 3.1%), compared to the nanosystems (Figure 4b) and the control treatment, but still demonstrated poor weed control (<85%). In *E. indica* plants, the commercial glyphosate provided higher control (89.2 ± 7.1%) than the ZPF3 formulation at 360 and 720 g a.e. ha^–1^ (38.3 ± 4.8 and 51.7 ± 12%, respectively) (Figure 5b).

Furthermore, it is possible to find glyphosate-sensitive *Ipomoea* sp. populations, where only 80 g a.e. ha^–1^ was sufficient to reduce the dry mass by 50% (GR_50_).^66^ Unsatisfactory results are often reported in the literature and observed in the field. Species like *I. purpurea* require high rates of glyphosate (1440–1925 g a.e. ha^–1^) to reduce the dry mass by 50%.^66^ For species such as *I. triloba*, glyphosate sequential application (960 g a.e. ha^–1^– 7 DAE and 480 g a.e. ha^–1^ – 14 DAE) led to control below 85% (50–77% of the population)^67^ and in populations of *I. grandifolia* studied by Pazuch et al.,^68^ a variation of 1000–3000 g a.e. ha^–1^ in glyphosate rates was necessary to reduce dry mass by 80% (GR_80_). This occurs due to the natural tolerance of *Ipomoea* sp. to glyphosate^69^ and some evidence points to differential translocation patterns.^70,71^ In addition, some researchers reported poor weed control of *E. indica* with glyphosate. In populations from Spain, glyphosate at 720 g a.e. ha^–1^ provided control of 68–73%;^72^ in China, the control at the same dose was 40–60% and 1440 g a.e. ha^–1^ was necessary to achieve 100% weed control;^73^ in Brazilian populations, doses of around 1080 g a.e. ha^–1^ provided 100% control in sensitive biotypes, whereas in the more tolerant biotypes, 2160 g a.e. ha^–1^ was necessary for satisfactory weed control.^74^

Considering the perspective of glyphosate toxicity improvement to *E. indica* and *I. grandifolia* plants, the nanoformulation was not able to improve this aspect (Figures 4 and 5) and the nanoformulation ZPF3 was not able to break the natural tolerance of *I. grandifolia* to glyphosate. Moreover, the mechanisms driving the interaction of ZPF3 and weed species require an in-depth investigation to enhance the knowledge concerning the safe-by-design approach in nanosystem development.

These results demonstrate the importance of weed species in evaluating nanoherbicide efficacy and that weed species alone are not enough to determine the applicability of these nanocarriers. However, it is important to mention that, in a scenario with *Amaranthus* spp. infestation, the glyphosate nanocarriers present potential for weed management (Section 3.3).

### 3.5 Nanoparticle/Plant Interaction

Nanoparticles have shown the potential to increase the toxicity of herbicides to target species. The mechanisms of interaction between nanoherbicides and plants are still being explored by the scientific community, mainly due to the expansion of possible combinations between polymers and herbicides and the plant species tested. For example, for metribuzin loaded with polymer particles, our group has characterized higher efficacy and biological response in *I. grandifolia*.^28^ Concerning interactions between nanosystem and plants, higher absorption of metribuzin in *Amaranthus viridis* was observed when associated with NPs, contributing to a higher toxicity.^75^ These effects can be related to nanomaterial characteristics (like surface area, amphiphilic characteristics, size, charge, and shape), which facilitate the particles to cross the cell wall and membranes,^76^ carrying a high amount of herbicide (known as the Trojan horse mechanism).^77^

**Figure 6:**
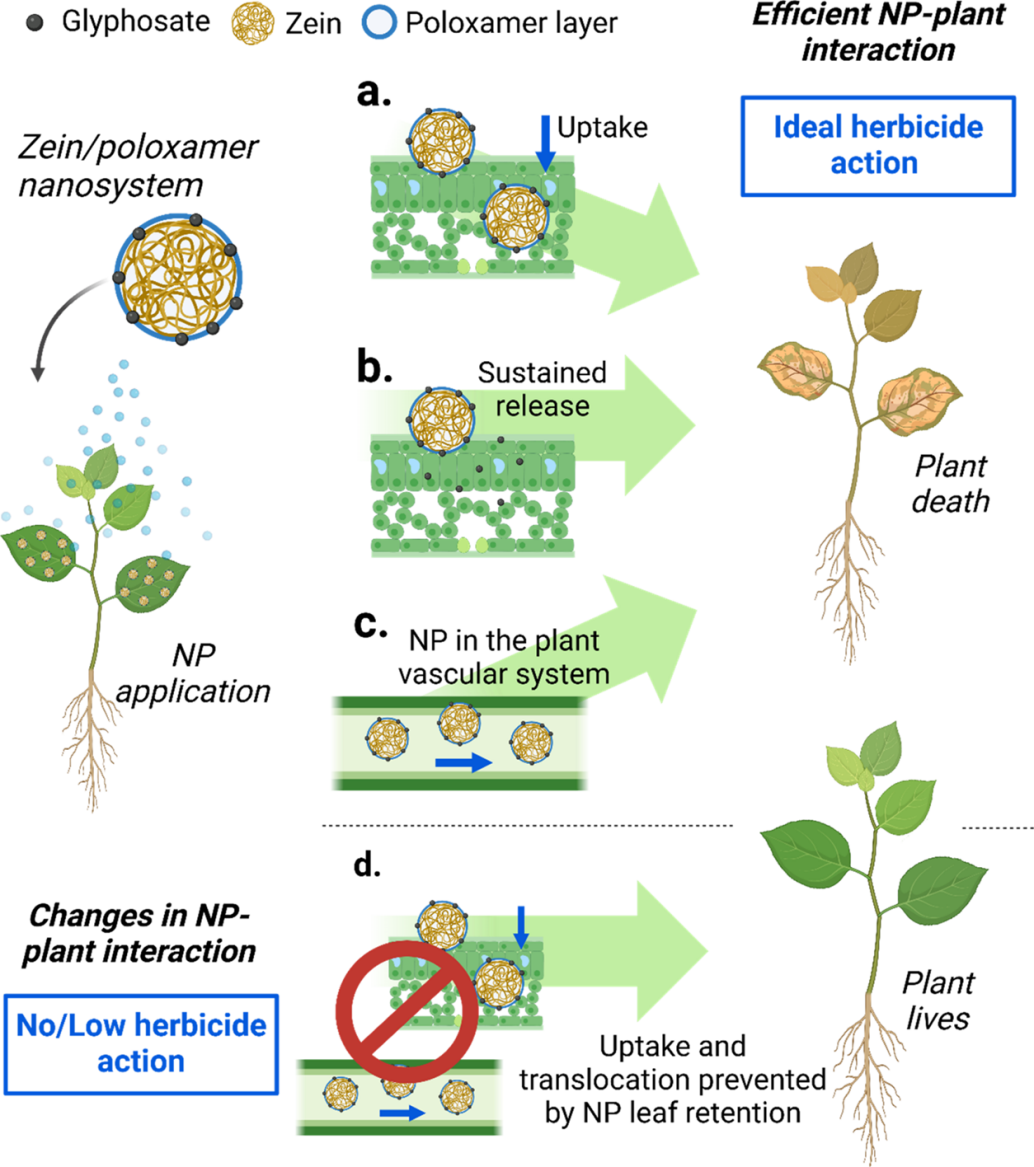
Schematic illustration of zein/poloxamer NP–plant interaction with *A. hybridus* plants after nanoherbicide application. Efficient NP–plant interaction, as ideal herbicide action, is represented by uptake increase (a), sustained release of herbicide (b), and translocation of NP in the plant vascular system (c), leading to plant death. Changes in the NP–plant interaction, such as no/low herbicide action, are represented by uptake and translocation, which are prevented by NP leaf retention, keeping the plant alive (d). Created with BioRender.

To date, three main hypotheses have been formulated to explain the higher activity of nanosystem-loaded glyphosate in *A. hybridus* plants (Figure 6a–c). In the initial interaction with plant leaves, nanosystems can enhance the retention and uptake of the herbicide, resulting in a higher concentration of glyphosate reaching the vascular system of the target species (Figure 6a). Furthermore, this may be attributed to a sustained-release mechanism, whereby the herbicide is released at varying rates and points within the plant system (Figure 6b). Similar results were observed in nanosystems for the delivery of metribuzin and atrazine.^27,75,78^ Nanomaterials have also been shown to increase the translocation of pesticides through plant vascular tissues,^79^ which can contribute to the enhanced efficacy of the herbicide^80^ (Figure 6c). In conclusion, nanoherbicides act multifaceted, enhancing herbicide efficacy primarily by altering herbicide–plant interaction.

The efficacy of nanoherbicides is contingent upon the specific target weed species, mainly due to the considerable intraspecies and intrapopulation variability observed in weed populations (Sections 3.3 and 3.4). This inherent complexity makes work with nanoherbicides more challenging. The findings indicate that the ZPF3 nanosystem exhibited inferior efficacy compared to the commercial glyphosate formulation of *E. indica*. The primary hypothesis for this outcome is based on the interactions depicted in Figure 5a–c. In cases where the plant is retaining the nanosystem on the leaf surface or within the tissues, the uptake and translocation of glyphosate nanosystems are prevented (Figure 6d). It can be confirmed that the association of glyphosate in the ZPF3 nanosystem did not increase the toxicity for *I. grandifolia*.

Furthermore, there is a change in the interaction between the nanosystem and the plants. This change is either minimal or occurs in the opposite direction (Figure 6d) compared to *A. hybridus* plants. In addition to these hypotheses, the mechanisms underlying these interactions remain to be elucidated and warrant further investigation.

### 3.6 Toxicity to Glyphosate-Tolerant Soybean and Cotton

**Figure 7:**
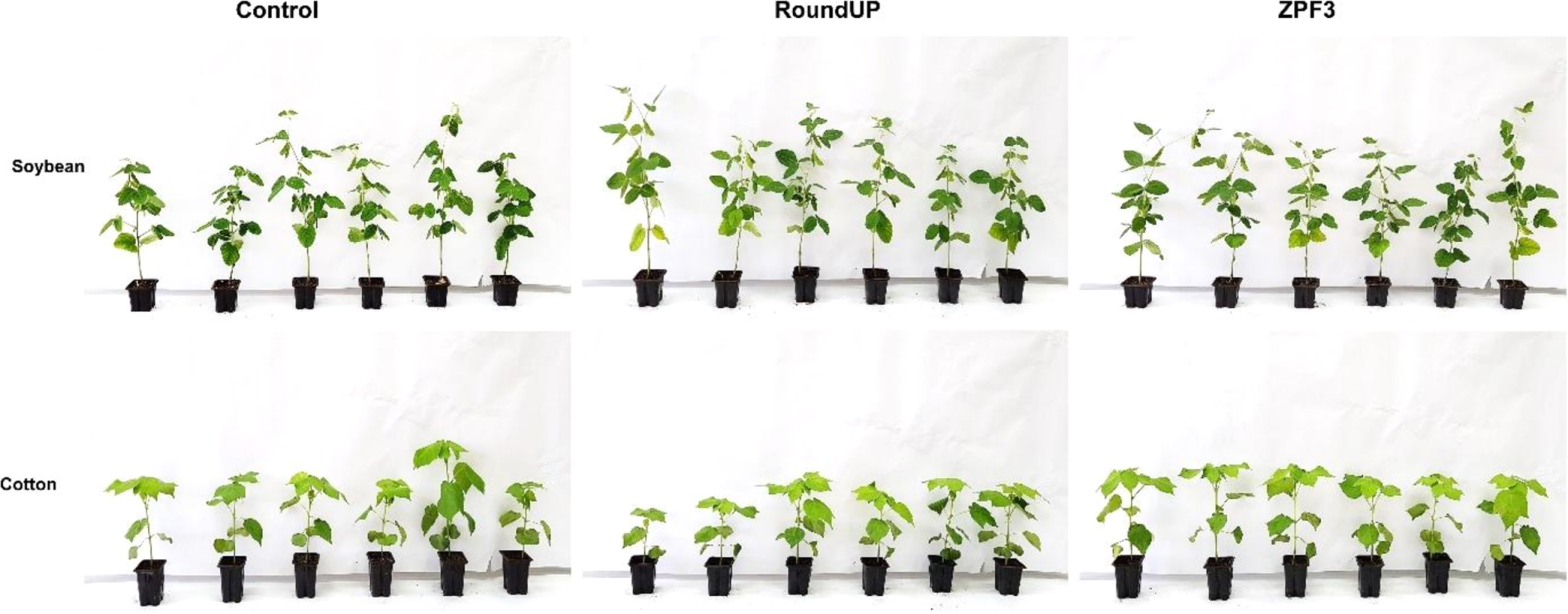
Tolerant soybean and cotton (RR crops) submitted to glyphosate application in different formulations, at 21 DAA. Self-explanatory.

Glyphosate formulations (ZPF3 and commercial) did not promote visual toxicity (Figure 7) to soybean and cotton plants, and the fresh weight at 21 DAA did not differ between the treatments (*p* > 0.05) (Table 3). Besides the effect of nanoparticles on the herbicide mode of action, which is still unknown, the most commonly reported effects of nanoparticles or nanoherbicides in plants occur in the interaction with the leaves and vascular systems.^27,78,81−85^ The nontoxicity of ZPF3 to glyphosate-tolerant crops was expected since glyphosate tolerance occurs due to the introduction of a cp4-gene that codifies an insensitive 5-enolpyruvylshikimate-3-phosphate synthase (EPSPS),^86^ and it is unlikely that the nanocarrier interferes in this process. The nontoxic effect on tolerant crops is important because it allows nanoformulation during the crop cycle, improving weed control without breaking the crop selectivity.

**Table 3:** Soybean and Cotton Fresh Weight (g) Submitted to Different Glyphosate Formulations

	fresh weight (g)^ns^	
treatments	soybean	cotton
control	10.8 ± 1	6.9 ± 1.9
roundUp–720 g a.e.^a^ ha^–1^	9.9 ± 1.8	7.2 ± 2
ZPF3–720 g a.e..ha^–1^	11.3 ± 1.1	8.1 ± 0.9

a a.e. ha^–1^ = Acid equivalent of glyphosate applied per hectare. *p*-value (Soybean) = 0.212 ^ns^*p*-value (Cotton) = 0.509^ns^

### 3.7 Effect on Soil Respiration

**Figure 8:**
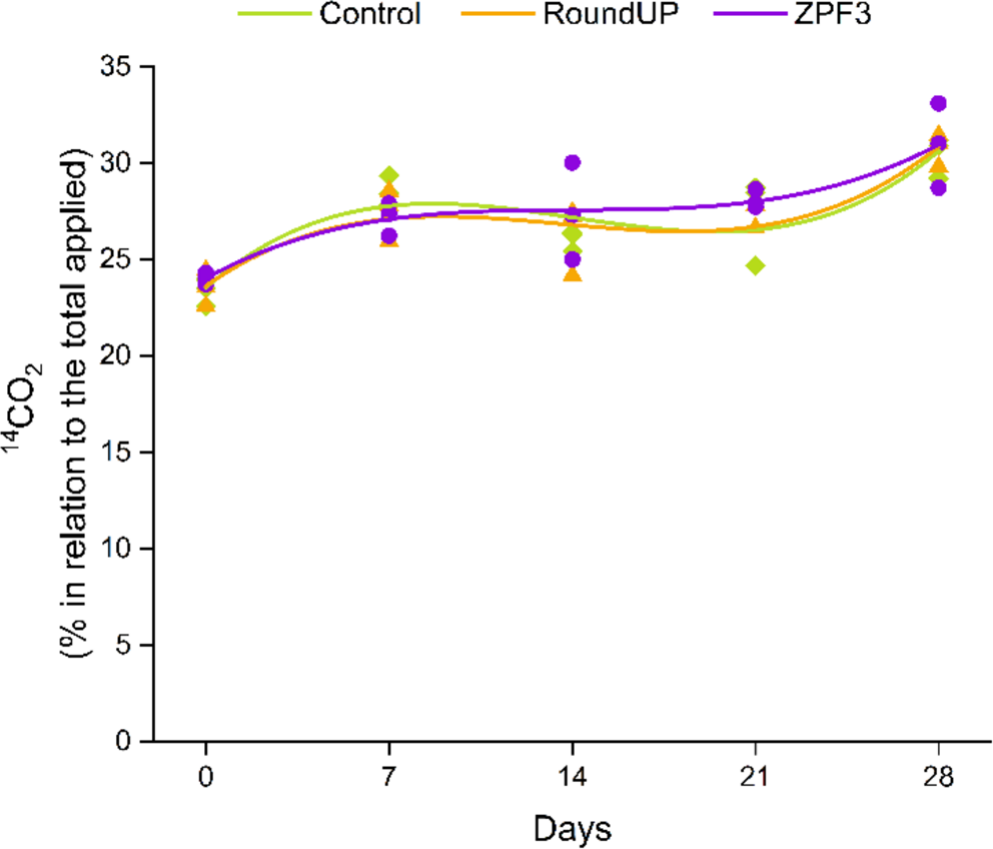
Mineralization of ^14^C-glucose from soil treated with different glyphosate formulations. The dots represent the data (*n* = 3), and the lines represent the adjustment for a cubic model regression (*y* = *ax*^3^ + *bx*^2^ + *cx* + *d*).

Nonsignificant effects in ^14^C-glucose mineralization were found between the treatments (*p* > 0.05) (Figure 8). A cubic model was adjusted to the data as a function of incubation time (Figure 7). Similar behavior was found in the curves of each treatment, indicating that RoundUp and ZPF3 did not promote a significant change in soil respiration compared to the control treatment over time (Figure 8). This occurs because soil microorganisms quickly degrade glyphosate and increase carbon mineralization.^87^ A meta-analysis reported a similar result: glyphosate stimulated microbial respiration for up to 60 days. Furthermore, it tends to decrease in the long term at levels lower than initial respiration (before glyphosate).^88^ Our results show no negative influence of RoundUp or ZPF3 on glucose mineralization in soil. However, this points to the need for further studies to understand the effect of glyphosate formulations on soil microbial activity and how nanoformulation can influence the microbial community in a long-term assay.

### 3.8 Effect on Soil Enzymes

**Figure 9:**
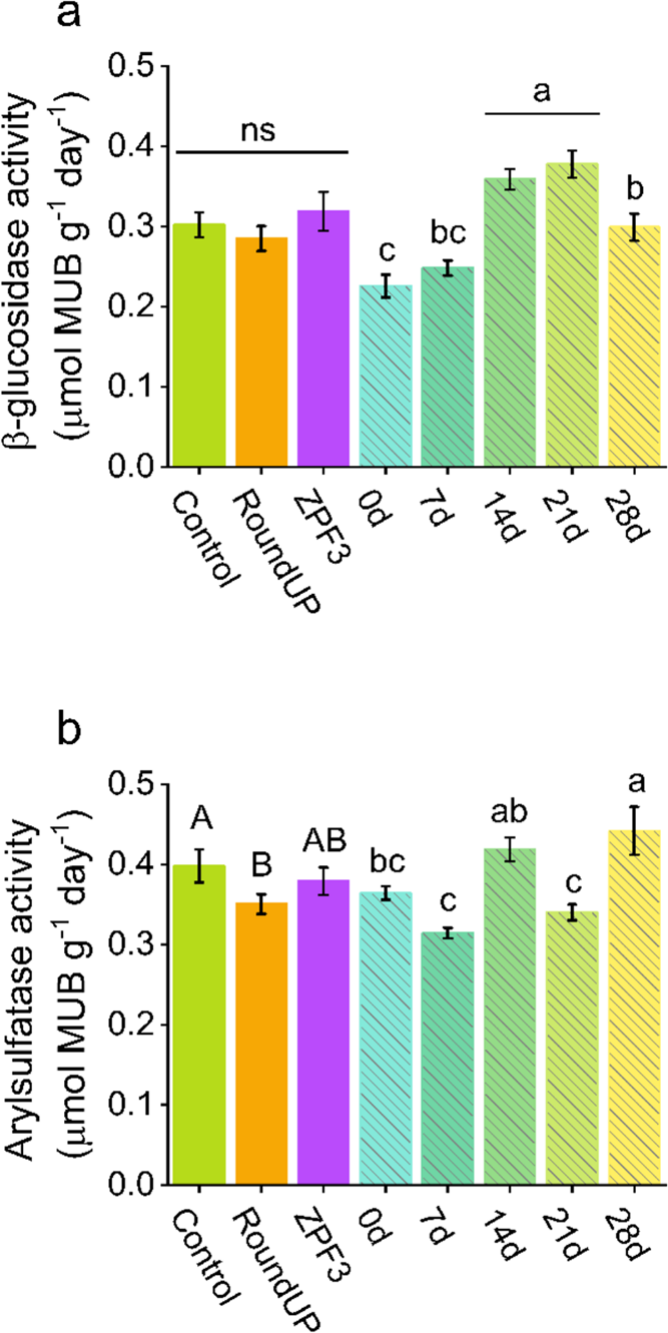
Soil enzyme activity of β-glucosidase (a) and arylsulfatase (b) submitted to different glyphosate formulations over 28 days. The data are mean ± standard error (*n* = 3). Uppercase letters represent differences between the factor formulation, and lowercase letters represent differences between the incubation period inside the same enzyme, by Tukey’s test (*p* < 0.05).

The enzyme activity results for β-glucosidase and arylsulfatase are presented in Figure 9. The evaluation time influenced both enzymes, whereas the formulation influenced only arylsulfatase (*p* < 0.05) (Figure 9). For β-glucosidase, the enzyme activity increased over time up to 21 DAA (0.22 ± 0.01 to 0.38 ± 0.02 μmol MUB g^–1^ day^–1^) but decreased at 28 DAA (0.29 ± 0.02 μmol MUB g^–1^ day^–1^) (Figure 9a), tending to return to initial equilibrium. The increase in activity after application can occur due to the rise in water, C, and P content in soil, since the herbicide treatments did not affect this enzyme.^89^ For arylsulfatase, commercial glyphosate (RoundUp) reduced by 11% (from 0.39 to 0.35 μmol MUB g^–1^ day^–1^) and ZPF3 provided an effect on enzyme activity similar to the control treatment and commercial glyphosate (Figure 9b). Higher enzyme activity was found in arylsulfatase at 14 DAA, which increased at 28 DAA (Figure 8b), different from that of β-glucosidase (Figure 9a).

According to Riah et al.,^90^ herbicides can be divided into a group with a few positive effects and another group that negatively affects the soil microbial community. Studies on the effects of glyphosate on soil enzymes and microbial communities indicate no negative effects,^91−93^ but some changes can be found. For example, the soil enzyme activity can be influenced by soil type and glyphosate doses; however, after 27 days, these effects can be reduced, as pointed out by Nguyen et al.^94^ Furthermore, the possibility of changing the microbial community over time and with repeated application is mentioned.^95,96^ Polymer-based nanosystems, such as nanometribuzin, reported by Takeshita et al.^28^ did not cause negative impacts on soil enzyme activity (such as β-glucosidase and arylsulfatase). However, other types of nanoparticles, such as magnetic carboxymethyl-β-cyclodextrin-Fe_3_O_4_ as carriers for diuron, can be toxic to the microbial community.^97^ The role of nanosystem design in the environmental safety of this new technology is highlighted herein for ZN/PL nanoparticles.

In summary, nanosystem development for glyphosate delivery can be performed using natural-based polymers, and an exploratory approach was needed to find the combinations within polymers and cross-linkers. The current study sheds light on the impact of nanoparticle design and weed species on the effectiveness of nanosystems, the potential for innovation in glyphosate formulation using natural-based polymers, and the need for a more pragmatic approach to developing practical nanosystems.

## Supporting Information Available

Analytical curve of glyphosate by LC-MS/MS, size distribution of the particles synthesized at first steps, tables with the volumes of each formulation used for solution preparation, and the data from the figures (PDF)
Data-glyphosate-manuscript (XLSX)
