# Supplementary material for: Nanobased Natural Polymers as a Carrier System for Glyphosate: An Interesting Approach Aimed at Sustainable Agriculture
Source: J Agric Food Chem. 2025 Jan 2;73(2):1097–111. doi: 10.1021/acs.jafc.4c08328 (PMC11741110; doi:10.1021/acs.jafc.4c08328)
Supplement: Supplementary file 1 — jf4c08328_si_001.pdf [file jf4c08328_si_001.pdf]

Supplementary information

Nano-based natural polymers as a carrier system for glyphosate: an interesting approach aimed at sustainable agriculture

Gustavo Vinícios Munhoz-Garcia[a*], Vanessa Takeshita[a,b], Jhones Luiz de Oliveira[b], Bruno Dalla Vecchia[c], Daniel Nalin[a], Camila de Werk Pinácio[a], Ana laura Camachos de Oliveira[c], Brian Cintra Cardoso[c], Valdemar Luiz Tornisielo[a], and Leonardo Fernandes Fraceto[b*].

[a]Center of Nuclear Energy in Agriculture, University of São Paulo, Av. Centenário 303, 13400-970, Piracicaba, SP, Brazil.

[b]Institute of Science and Technology, Sao Paulo State University, Av. Três de Março, 511 - Alto da Boa Vista, 18087-180, Sorocaba, SP, Brazil.

[c]Superior School of Agriculture "Luiz de Queiroz", University of São Paulo, Av. Pádua Dias, 11, 13418-900, Piracicaba, SP, Brazil.

*Corresponding authors. Emails: gvmgarcia@usp.br, leonardo.fraceto@unesp.br

Tables

Table S1. Application solutions are used in biological assays for plants of A*maranthus hybridus*. The final volume is 7 mL, consisting of 6 pots + 1 extra pot.

Treatment	Concentration (mg a.e. mL^{-1})	Volume	
		Formulation (µL)	Water (mL)
Control (Without herbicide)	0	0	7
RoundUp – 720 g a.e*. ha^{-1}	360	5.38	7
ZLF2 – 720 g a.e. ha^{-1}	1.66	1167	5.83
ZLF2 – 360 g a.e. ha^{-1}	1.66	584	6.41
ZPF1 – 720 g a.e. ha^{-1}	1.66	1167	5.83
ZPF1 – 360 g a.e. ha^{-1}	1.66	584	6.41
ZPF2 – 720 g a.e. ha^{-1}	2.49	778	6.22
ZPF2 – 360 g a.e. ha^{-1}	2.49	389	6.61
ZPF3 – 720 g a.e. ha^{-1}	3.32	584	6.41
ZPF3 – 360 g a.e. ha^{-1}	3.32	292	6.71

*a.e. ha^{-1}= acid equivalent of glyphosate per hectare

Table S2. Application solutions are used in biological assays for plants of *Eleusine indica* and *Ipomoea grandifolia*. The final volume is 7 mL and consists of 6 pots + 1 extra pot.

Treatment	Concentration (mg a.e. mL^{-1})	Volume	
		Formulation (µL)	Water (mL)
Control (Without herbicide)	0	0	7
RoundUp – 720 g a.e*. ha^{-1}	360	5.38	7
ZPF3 – 720 g a.e. ha^{-1}	3.32	584	6.41
ZPF3 – 360 g a.e. ha^{-1}	3.32	292	6.71

*a.e. ha^{-1}= acid equivalent of glyphosate per hectare

Figures

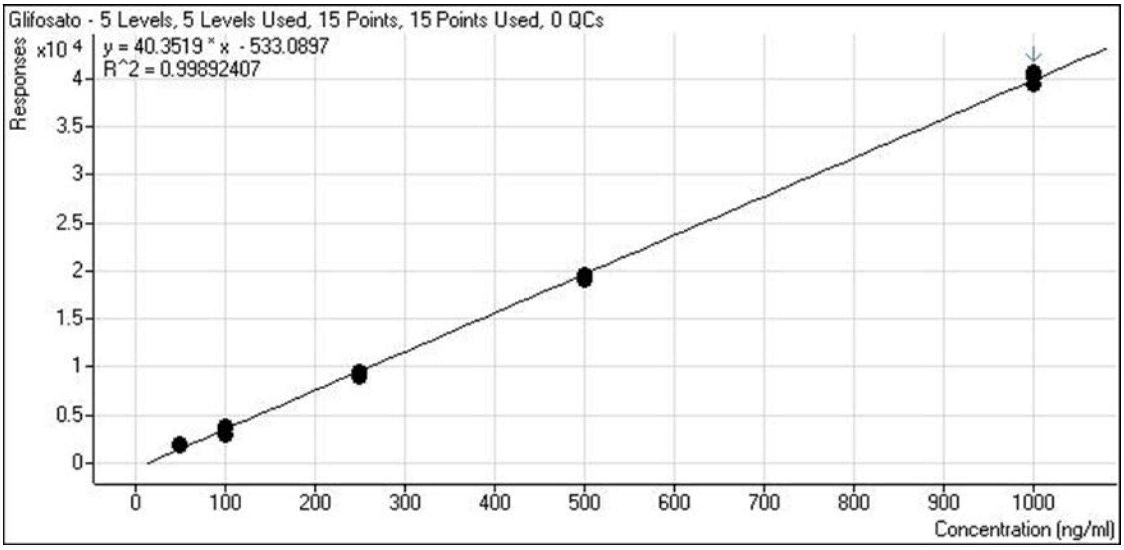

Figure S1. Analytical curve for glyphosate detection by LC-MS/MS. The dots represent the peak area obtained in the chromatography in function of the standard concentration in each stock solution (n = 3).

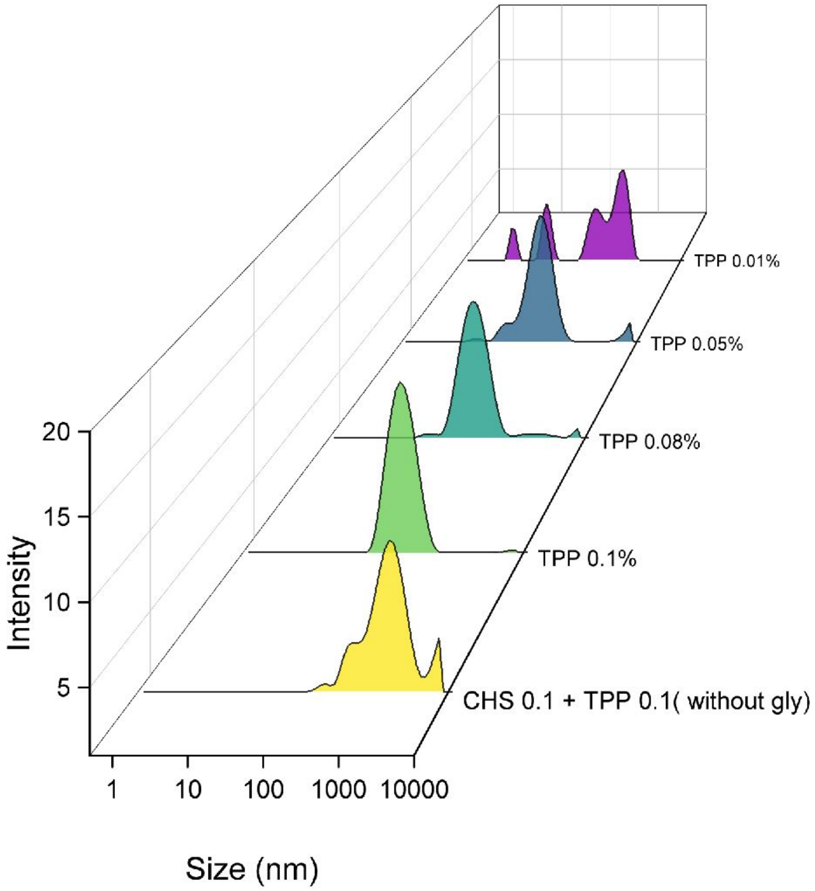

Figure S2. Particle size distribution (measured by DLS) of nanoformulations based on chitosan/TPP containing glyphosate (1.5 mg mL^{-1}), under different TPP concentrations.

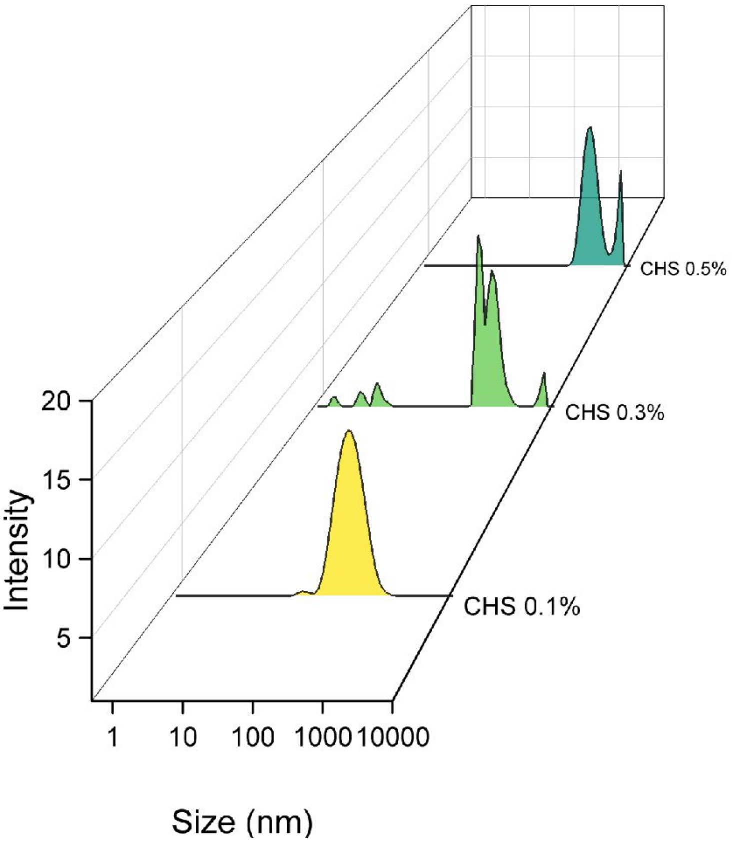

Figure S3. Particle size distribution (measured by DLS) of nanoformulations based on chitosan/TPP containing glyphosate (1.5 mg mL^{-1}), under different chitosan concentrations.

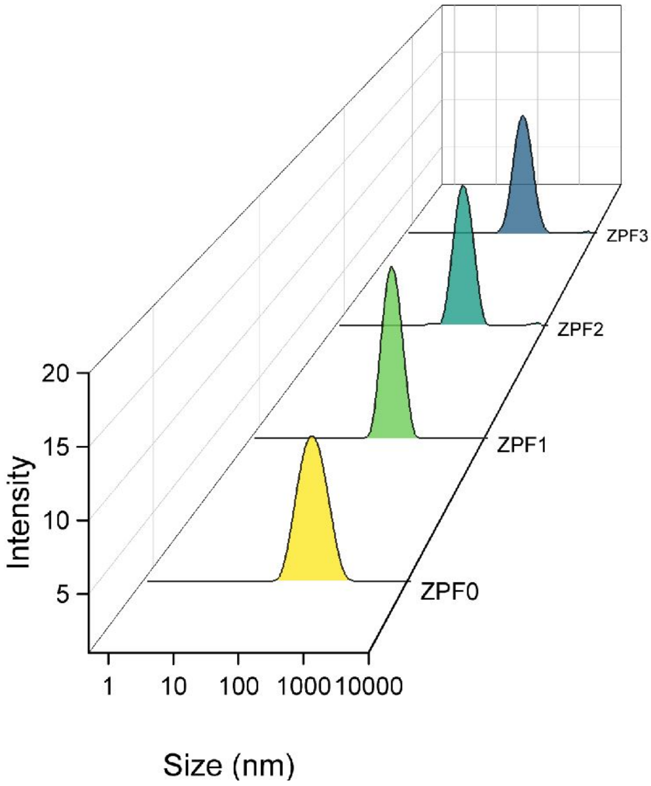

Figure S4. Particle size distribution (measured by DLS) of nanoformulations based on zein/poloxamer containing glyphosate in different concentrations (2 - 4 mgmL^{-1}). ZPF0 - zein/poloxamer, ZPF1 – zein/poloxamer 2 mg mL^{-1}, ZPF2 – zein/poloxamer 3 mg mL^{-1}, ZPF3 – zein/poloxamer 4 mg mL^{-1}.

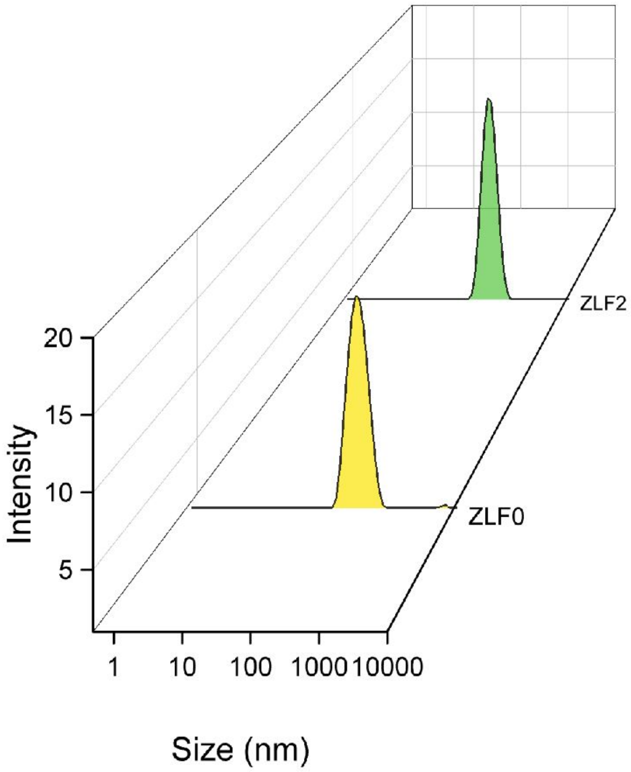

Figure S5 Particle size distribution (measured by DLS) of nanoformulations based on zein/lignin containing glyphosate in different concentrations (0 and 2 mg mL^{-1}). ZLF0 - zein/lignin, ZLF2 – zein/lignin 2 mg mL^{-1}.

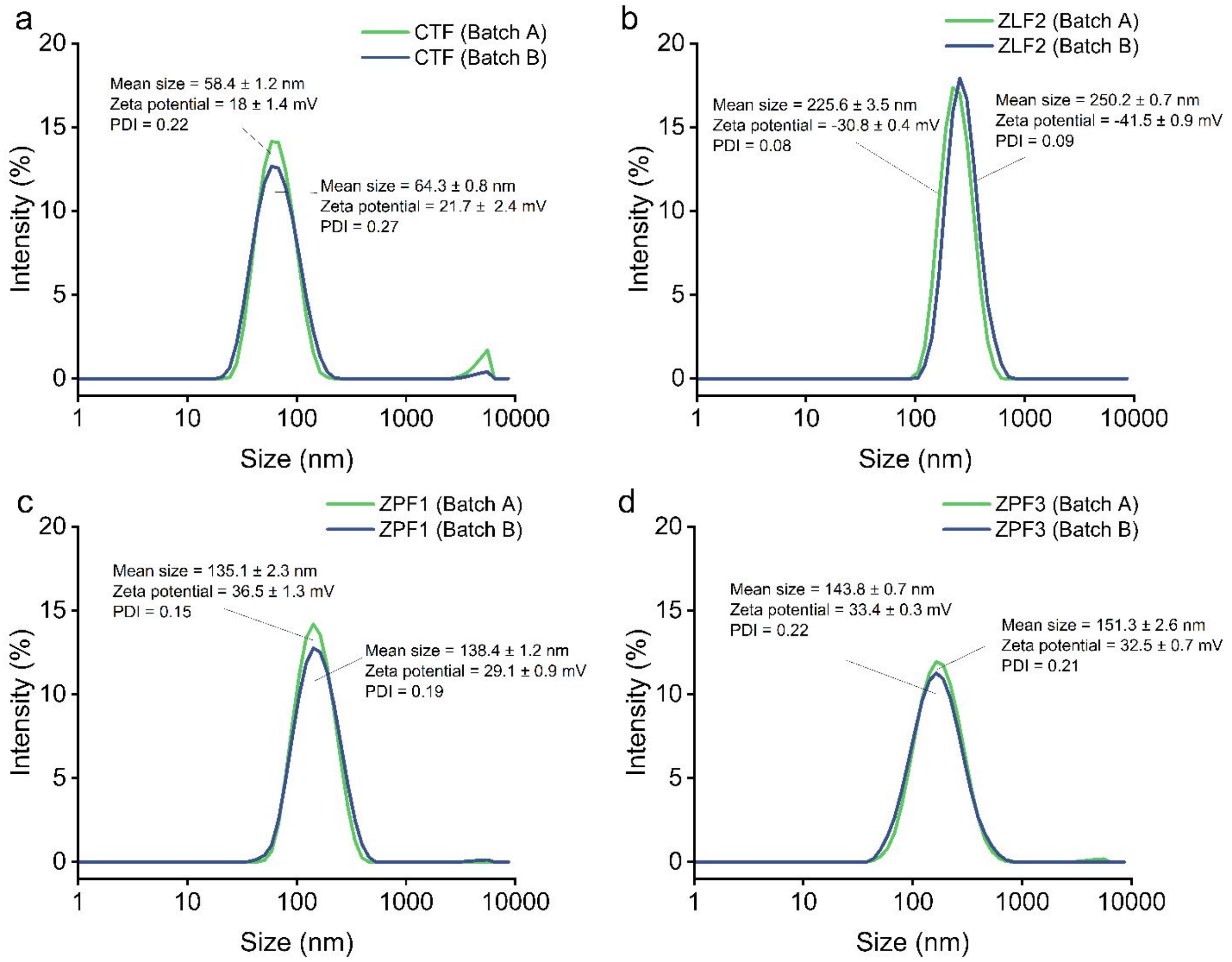

Figure S6. Particle size distribution (measured by DLS) of nanoformulations based on chitosan/TPP, zein/lignin, zein/poloxamer, produced in different batches.

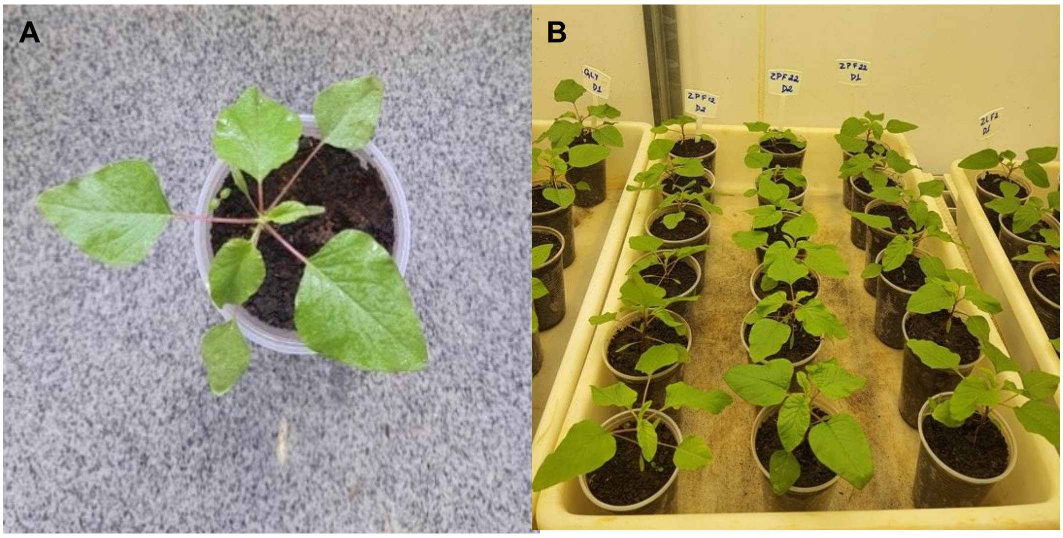

Figure S8. (A) *Amaranthus hybridus* plant after solution application with manual spray and (B) experimental unity in the growing chamber after application.
